# Biochemical and biophysical characterization of purified native CD20 alone and in complex with rituximab and obinutuzumab

**DOI:** 10.1038/s41598-019-50031-4

**Published:** 2019-09-23

**Authors:** Morgane Agez, Elodie Desuzinges Mandon, Thomas Iwema, Reto Gianotti, Florian Limani, Sylvia Herter, Ekkehard Mössner, Eric A. Kusznir, Sylwia Huber, Matthias Lauer, Philippe Ringler, Claudia Ferrara, Christian Klein, Anass Jawhari

**Affiliations:** 1 CALIXAR, 60 avenue Rockefeller 69008, Lyon, France; 2 Roche Pharma Research & Early Development, Roche Innovation Center Zurich, Schlieren, Switzerland; 3 Roche Pharma Research and Early Development, Lead Discovery, Roche Innovation Center Basel, Basel, Switzerland; 4 Center for Cellular Imaging and NanoAnalytics (C-CINA), Biozentrum, University of Basel, Basel, Switzerland

**Keywords:** Membrane proteins, Antibody therapy, Antibody therapy, Atomic force microscopy, Cancer immunotherapy

## Abstract

CD20 is a B-lymphocyte specific integral membrane protein, an activated-glycosylated phosphoprotein expressed on the surface of B-cells and a clinically validated target of monoclonal antibodies such as rituximab, ocrelizumab, ofatumumab and obinutuzumab in the treatment of all B cell lymphomas and leukemias as well as autoimmune diseases. Here, we report the extraction and purification of native CD20 from SUDHL4 and RAMOS cell lines. To improve the protein yield, we applied a calixarene-based detergent approach to solubilize, stabilize and purify native CD20 from HEK293 cells. Size Exclusion Chromatography (SEC) and Analytical Ultracentrifugation show that purified CD20 was non-aggregated and that CD20 oligomerization is concentration dependent. Negative stain electron microscopy and atomic force microscopy revealed homogenous populations of CD20. However, no defined structure could be observed. Interestingly, micellar solubilized and purified CD20 particles adopt uniformly confined nanodroplets which do not fuse and aggregate. Finally, purified CD20 could bind to rituximab and obinutuzumab as demonstrated by SEC, and Surface Plasmon Resonance (SPR). Specificity of binding was confirmed using CD20 antibody mutants to human B-cell lymphoma cells. The strategy described in this work will help investigate CD20 binding with newly developed antibodies and eventually help to optimize them. This approach may also be applicable to other challenging membrane proteins.

## Introduction

CD20 is highly expressed on the cell surface of B-cell lymphocytes and is a membrane marker of malignant B cells. CD20 is a member of the MS4A (multispanning 4A) family and is the best studied member of this family^1^. The protein has no known natural ligand and its function is poorly understood, even though evidence from humans suggest that CD20 is important to T-cell independent antibody responses^2^. It was suspected to act as a calcium channel in the cell membrane^3^, but since then this role has not been established. CD20 was shown to form homo-tetramers that physically associate with the B-cell antigen receptor^4^. The 35 kDa integral membrane protein consists of large, intracellular, amino and carboxyterminal domains connected to 4 transmembrane helices. The cytoplasmic domains are heavily phosphorylated at serine and threonine residues^5–10^. The extracellular part of CD20 consist of two loops from position 72 to 80 and from 142 to 182. These extracellular loops are well targeted by specific CD20 therapeutic monoclonal antibodies such as ofatumumab, rituximab, ocrelizumab and obinutuzumab^11,12^. Rituximab is a monoclonal anti-CD20 antibody that pioneered cancer therapy, since it was the first antibody to be approved for the treatment of lymphoma, representing one of the most important advances in the treatment of lymphoproliferative disorders in the last 40 years^13^. It was subsequently approved for non-Hodgkin lymphoma (NHL) and chronic lymphocytic leukemia (CLL) treatments^14^. Rituximab, ocrelizumab and ofatumumab are Type I CD20 antibodies whereas obinutuzumab is a Type II CD20 antibody. Obinutuzumab is a humanized Type II anti-CD20 antibody that has been glycoengineered to enhance FcγRIIIa interactions^15^. Obinutuzumab binds to the surface of CD20 at lower density than rituximab, a characteristic feature of Type II CD20 antibodies^15,16^. After binding to the CD20 expressing cells, antibodies are believed to trigger at least three different functions: Cell death, antibody-dependent cellular cytotoxicity and phagocytosis (ADCC/ADCP) as well as complement-dependent cytotoxicity (CDC)^13,17^.The different behavior of Type I and Type II CD20 antibodies is surprising because both antibodies recognize a partially overlapping epitope of CD20. Previous crystallographic studies have helped CD20 epitope characterization and could provide clues about Type I and Type II antibodies recognition^16^. These crystal structures relied on an epitope cyclic CD20 peptide that mimics the large extracellular loop of CD20. Despite this progress, a complete picture of full-length CD20 antibodies recognizing CD20 in term of spatial arrangements, binding features and their contribution to understand the mechanism of action, remains unclear. Molecular understanding of the interactions of CD20 with antibodies, requires the production and isolation of native CD20 protein. Here we report the purification of CD20 from plasma membranes of cancer cells (SUDHL4 and RAMOS). We show that recombinant wildtype CD20 can be solubilized and purified using a novel class of calixarene based detergents to solubilize and purify non-aggregated CD20. Previous reported work used peptidic construct, GST fusion CD20 for purification or *E.coli* for the expression^16,18^. Using a new class of detergent, we were recently able to produce and characterize homogenous and functional membrane proteins such as GPCR, ion channels and transporters^19–23^. This class of detergents was also helpful for maintaining protein/ protein interactions of BAG3 pancreatic cancer marker while solubilizing its corresponding membrane protein partner at the surface of macrophages, allowing its identification by mass spectrometry^24^. Calixarenes based detergents (Fig. S3) are believed to be useful for extracting and stabilizing membrane proteins, by allowing an efficient competition with lipids due to the hydrophobic tail, by establishing electrostatic interactions with membrane proteins residues thanks to the carboxylate groups and finally by making π-stacking interactions with aromatic residues. In addition to this, the glycosylated amphiphilic calixarene based detergent, has mild glycosides groups with stabilizing features (Fig. S3B)^25–27^. Here, we could solubilize, stabilize, purify native human CD20 and further characterize it in solution by size exclusion chromatography (SEC), analytical ultracentrifugation (AUC), electron microscopy (EM) & atomic force microscopy (AFM). Rituximab and obinutuzumab bound to purified CD20 in a surface plasmon resonance (SPR) experiment. The specificity of the binding was confirmed by SPR using purified CD20 and human B-cell lymphoma cells. Different CD20 antibodies of different affinities were used.

## Results

### Expression and purification of CD20 from SUDHL4 and RAMOS cells

**Figure 1:**
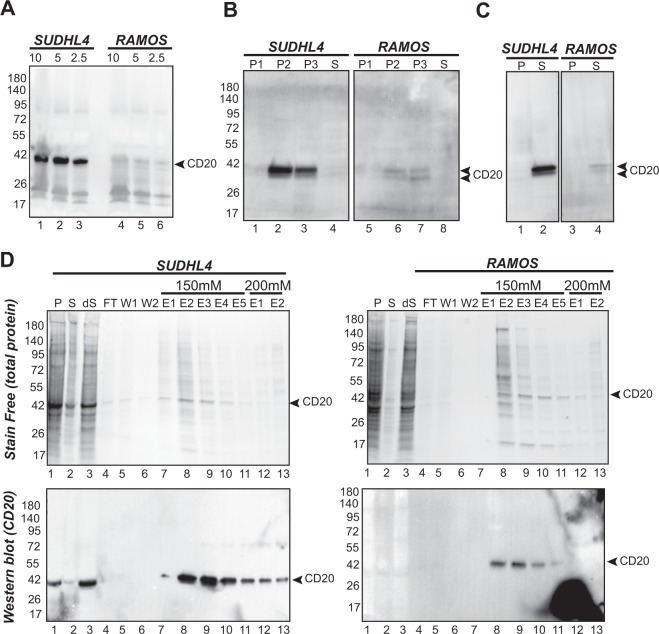
Expression and CD20 enrichment from SUDHL4 and RAMOS cells. (**A**) CD20 expression. Cells were harvested from 5 ml of culture medium at 1000 g for 5 min and lysed in PBS + laemmli buffer (1X final). Proteins from cell lysates (10 µl, 5 µl and 2,5 µl, corresponding to 3, 1.5 and 0.75 10^5^ cells respectively) were separated on a 4–15% Tris-glycine SDS-PAGE, transferred to PVDF membrane and immunodetected with an anti-CD20 (1:400) primary antibody and an anti-IgG horseradish peroxidase conjugated secondary antibody (3:10000). (**B**) Membrane fractionation of SUDHL4 and RAMOS cells expressing CD20. Fractions generated by membrane fractionation of SUDHL4 and RAMOS cells were loaded in a 4–15% Tris Glycine gel (10 µg of total protein for each fraction) and proteins separated by SDS-PAGE (4–15%). The western blot was probed with an anti-CD20 primary antibody (1:200) followed by an anti-IgG horseradish peroxidase conjugate secondary antibody (3:10000). P1, pellet after centrifugation at 1000 g; P2, pellet after centrifugation at 15000 g; P3, pellet after centrifugation at 100000 g (plasma membrane) S, supernatant after centrifugation at 100000 g. (**C**) Extraction of CD20 from both SUDHL4 and RAMOS cells. Plasma membranes from SUDHL4 and RAMOS cells were solubilized with 1% FC12. After extraction, soluble and insoluble fractions were separated by centrifugation. Proteins from each fraction were separated by SDS-PAGE. The western blot was probed with an anti-CD20 primary antibody (1:200) followed by an anti-IgG horseradish peroxidase conjugate secondary antibody (3:10000). P, pellet; S, supernatant. (**D**) Enrichment of CD20 using ion exchange chromatography DEAE after solubilization of SUDHL4 and RAMOS plasma membranes. Proteins from pellets (P), supernatants (S), diluted supernatant (dS) flow through (FT), wash (W), elution 1 to 5 (E1 to E5 using 150 mM NaCl) and then elution 1 and 2 (E1 and E2 using 200 mM NaCl) were separated on a 4–15% acrylamide SDS-PAGE. Total proteins were detected after stain-free activation (BioRad method). The presence of CD20 in each fraction was immunodetected with a mouse anti-CD20 (Abcam, 1:200) as a primary antibody and an anti-mouse IgG HRP conjugate as a secondary antibody (3:10000). Full-length gels and blots are included in a Supplementary Information file (Fig. S1).

To isolate and characterize native CD20, we decided to work on SUDHL4 and RAMOS cancer cell lines since they both naturally express CD20 (www.atcc.org). Indeed, expression was confirmed by western blot Fig. 1A. SUDHL4 cells showed a higher expression of CD20 in comparison to RAMOS. After cell lysis, membranes were prepared by sequential centrifugation at 1000G (P1), 15000G (P2) and 100000G (P3) to allow separation of cell debris, enriched ER/mitochondrial membranes and plasma membranes, respectively. Pellets obtained after each centrifugation (P1, P2 and P3) as well as the final soluble fraction were analyzed by SDS-PAGE and immunodetected by an anti-CD20 antibody. CD20 was present in P2 and P3 fractions (Fig. 1B, lane 2, 3 for SUDHL4 and 6, 7 for RAMOS) and was successfully solubilized from both cell lines (Fig. 1C, compare supernatant (S) lane 2 and 4 to the pellet (P), lanes 1 and 3). A better signal of CD20 was observed for SUDHL4 fractions when compared to RAMOS. This is consistent with expression tests (Fig. 1A) and FACS analysis (data not shown). After significant optimization of pH and salt concentrations, CD20 could be purified from SUDHL4 and RAMOS solubilized plasma membranes using DEAE chromatography. Figure 1D shows that CD20 was mainly recovered at 150 mM NaCl in fraction E2 to E4 for both cell lines as illustrated by western blot and stain free gels. Although CD20 was significantly enriched and specifically eluted from the column, a significant number of contaminants were still present in the samples. For this reason, we decided to recombinantly express a His-tag version of the full length and wild type CD20 (UniProt P11836) to allow affinity purification and therefore improve purity.

### Expression and purification of recombinant full length and wild type CD20

**Figure 2:**
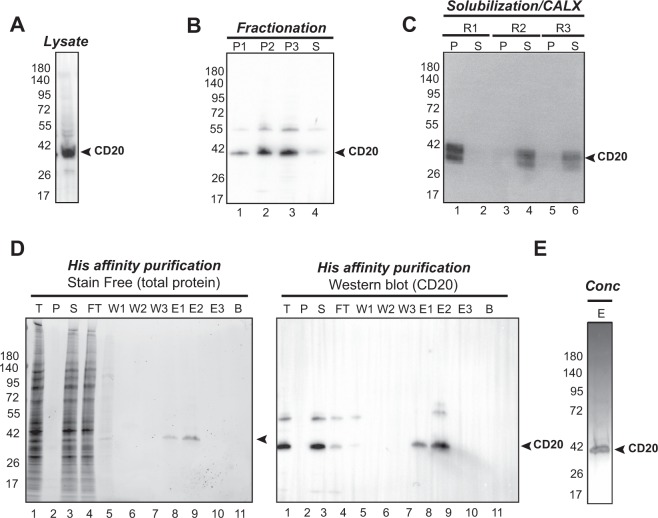
Expression and CD20 purification from HEK293 cells. (**A**) Expression of CD20 in HEK293 cells assessed by SDS-PAGE, transferred to PVDF membrane and immunodetected with an anti-His-HRP antibody (3:2000). (**B**) Membrane fractionation of HEK293 cells expressing CD20 and analyzed by western blot. P1, P2, P3 and S correspond to 1000 G centrifugation (cell debris), 15 000 G centrifugation (internal membranes), 100 000 G centrifugation (plasma membranes) and S (supernatant of the last ultracentrifugation), respectively. (**C**) CD20 solubilization trials using CALX-reagents R1, R2 and R3. CD20 was extracted from enriched plasma membranes fraction (P3, shown in Fig. 2B, lane 3) using 10 Critical Micellar concentration (CMC) of detergent. P and S correspond to non-solubilized (pellet) and solubilized fractions, respectively. CD20 presence was analyzed by western blot. (**D**) Affinity purification of CD20. CALX-R2 solubilized CD20 was purified by His-affinity and analyzed by western blot. Protein fractions from the Total (T), Pellet (P), Supernatant (S), Flow-Through (FT), Wash (W), Elution (E) and beads (B) after elution were analyzed by western blot. (**E**) Concentrated CD20 analyzed by western blot. W1–3 corresponds to washing steps of 10 column volume. E1-3 corresponds to elution steps using 1 column volume. Full-length gels and blots are included in a Supplementary Information file (Fig. S2).

To express CD20 in HEK293 cell, the plasmid pETR17026 containing CD20 cDNA was transfected using Polyethylenimine (PEI) transfection reagent. Expression in HEK293 cells was performed at 37 °C as described in Material & methods. A series of expression tests were performed in order to determine the condition with the highest expression. To this aim, three parameters were evaluated: time of post-transfection, DNA amount and the ratio DNA/ PEI. Expression of CD20 was detected by western blot using an anti-His antibody. A band at ~42 kDa corresponding to the expected size of the protein was detected (Fig. 2A). The condition showing the highest expression rate used 4.5 µg DNA and 9 µg PEI for transfection over 48 H. This condition was reproduced at a larger scale (1 L) of HEK293 cells from which membranes were prepared by sequential centrifugation as described above and analyzed by SDS-PAGE followed by immunodetection by an anti-His antibody. CD20 was mainly detected in both internal and plasma membrane fractions (Fig. 2B, lane 2 and 3). This was also observed for SUDHL4 and RAMOS cells. CD20 extraction tests were carried out using 3 different calixarene based detergents. Figure 2C shows that while CALX-R1 did not allow CD20 solubilization, CALX-R2 and CALX-R3 could efficiently solubilize the protein with a slightly better yield for CALX-R2 (>90%). Therefore, CALX-R2 described in Fig. S3 was applied for further CD20 purification steps. Solubilized proteins were then loaded on a Nickel His-affinity column. Non-specifically bound proteins were removed with intensive washes using 50 mM Imidazole, 500 mM NaCl buffer. Elution of CD20 was achieved using 500 mM Imidazole. Samples from each fraction were then analyzed by SDS-PAGE followed by immunodetection with the anti-His antibody. Specific binding and elution of CD20 were observed on both SDS-PAGE stain free gel as well as western blot (Fig. 2D). The purity of CD20 was much higher than that obtained from SUDHL4 and RAMOS cells (~95% instead of ~50%) as shown in diluted and concentrated pure CD20 in Fig. 2D, lane 9 and even after concentration of the purified protein **(**Fig. 2E**)**.

### Behavior of purified native CD20 in solution

To assess the behavior of purified CD20 in solution, we concentrated the protein and loaded it on a Superose 6 Gel filtration column equilibrated with CALX-173-GK instead of CALX-R2 to be compatible with surface plasmon resonance. In contrast to CALX-R2 which exhibits very good solubilization properties, CALX-173-GK (described in Fig. S3) does not solubilize membrane proteins very well. CALX-173-GK was recently reported to stabilize functional membrane proteins^27^. In addition to that, CALX-R2 may interfere with Ni-NTA chip immobilization of proteins in contrary to CALX-173-GK^28^. For these reasons, we decided to combine both features of the two molecules to allow effective solubilization/ purification and downstream SPR immobilization of CD20.

**Figure 3:**
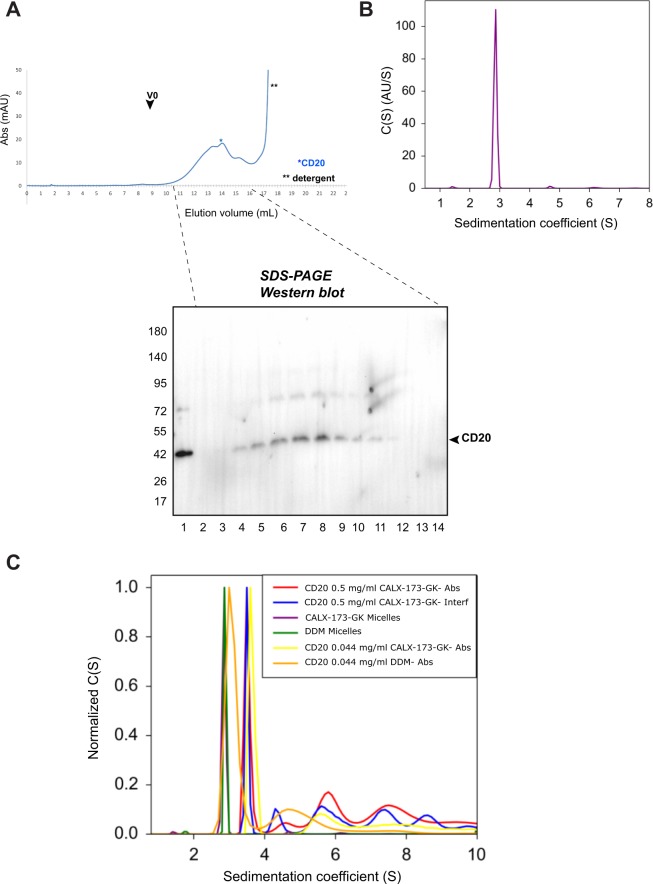
Behavior in solution of purified CD20. (**A**) Gel filtration chromatography (Superose 10/300 Increase) on purified CD20. Chromatogram is shown in Top. Protein fractions were analyzed by SDS-PAGE and western blot (Bottom). Vo indicates the void volume of the column. * and **indicates CD20 and detergent elution volume, respectively. CALX-173-GK detergent absorbs at 280 nm. Full length SEC chromatogram is shown in Fig. S4. (**B)** Distribution of sedimentation coefficient c(s) for the CALX-173-GK micelles at 0.5% (w/v) detergent. Interference and absorbance detection (250 nm) are shown. (**C**) Normalized distribution of sedimentation coefficient c(s) for CD20 protein (0.044 and 0.5 mg/ml) and detergent micelles (0.5% CALX-173-GK and 0.05% (w/v) DDM) with absorbance (250 nm) and interference detection. Full-length gels and blots are included in a Supplementary Information file (Fig. S2).

The elution chromatogram of CD20 (Fig. 3A) showed a profile of a non-aggregated protein with no signal in the void volume of the column. CD20 was detected by western blot within a broad peak (indicated by *). To have a more precise assessment of CD20 species in solution, sedimentation coefficient distribution profile was determined in AUC sedimentation velocity (SV) experiment. Prior to the analysis of detergent solubilized CD20 protein, the control sample containing the CALX-173-GK detergent at 0.5% (*w/v*) (above its critical micelle concentration) was analyzed. The SV experiment showed monodisperse species with a signal weighted sedimentation coefficient sw of 2.9S which correspond to the detergent CALX-173-GK micelle (Fig. 3B). The aggregation number of CALX-173-GK in the micelle was 27 as calculated from the molecular weight (38400 Da) determined for the detergent micelle alone in the SV experiment (Table S9). The CD20 protein sample (at 0.5 mg/ml) shows polydispersity as observed in the AUC SV experiment. The most dominant species were sedimenting with a signal weighted sedimentation coefficient sw of 3.5S corresponding presumably to the monomeric, detergent solubilized CD20 protein. Sedimenting species with higher signal weighted sedimenting coefficients (sw >4S) were observed next to monomeric CD20 and correspond to higher molecular weight oligomers. Interestingly, dilution of the CD20 protein to a concentration of 0.044 mg/ml lead to changes in the sedimentation coefficient distribution profile (Fig. 3C). This result suggests a CD20 concentration dependent oligomerization. Sedimenting species corresponding to monomeric detergent solubilized CD20 (sw of 3.5 S) were more present in the diluted sample (Fig. 3C). The exact quantification of absorbance signal intensities corresponding to distinct CD20 species was limited, as both, the protein itself and the CALX-173-GK detergent contributed to the absorbance signal. Nevertheless, the contribution of the detergent to the absorbance signal at the applied detergent concentration (0.072%) was much lower than protein absorbance. Thus, qualitatively it can be stated, based on the sedimentation data that a clear shift in the sedimentation coefficient distribution profile is observed in favor of the monomeric detergent solubilized CD20 species (sw of 3.6S, Table S9) for the diluted protein sample. A similar effect was noticed by dilution of the concentrated CD20 sample in a buffer containing n-Dodecyl-β-D-maltoside (DDM) detergent (0.05%) where the most likely monomeric CD20 species sedimented with slightly lower signal weighted sedimentation coefficient sw of 3.1S.

**Figure 4:**
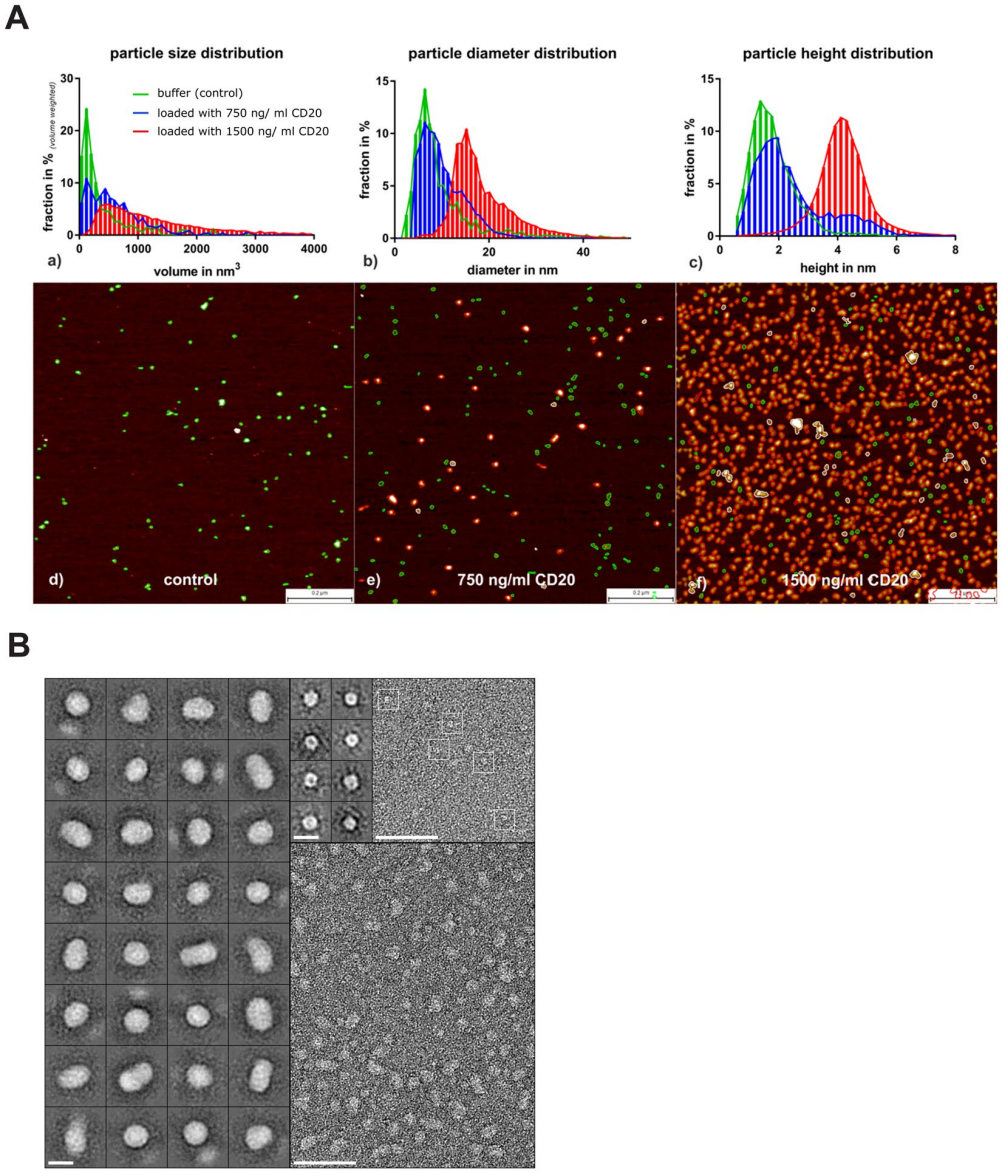
CD20 analysis by EM and AFM. (**A)** Morphology data recorded with tapping mode AFM for CD20 in micellar solution of CALX-173-GK on the surface of mica. Particle volume distribution profiles of micellar particles in the absence and presence of CD20 (green: without CD20, blue: with c_CD20_ = 750 ng/ml, red: with c_CD20_ = 1500 ng/ml). Fig b and c) show the corresponding diameter and height distribution profiles (see color legend in a). Figure d,e) show AFM height maps: green particles match the morphology and size of the green population and red particles those of red populations in a–c). (**B**) Size and shape of CD20 and CALX-173-GK micelles as observed by negative staining transmission electron microscopy. Left panel: gallery of 32 class averages of CD20 particles (around 150 particles averaged per class), scale bar = 10 nm. The round and ovoid particles have sizes ranging from 9 nm to 15 nm Bottom right panel: raw micrograph of negatively stained CD20 preparation, scale bar = 50 nm. Top right insert: at left, gallery of 8 class averages of CALX-173-GK detergent micelles (around 50 particles averaged per class), the micelles have a diameter of 5–6 nm, scale bar = 10 nm; at right, raw micrograph of negatively stained CALX-173-GK buffer, some particles are marked with white boxes, scale bar = 50 nm.

We next decided to analyze the CD20 preparation using Atomic Force Microscopy (AFM). The control sample which contains the detergent above its critical micellar concentration showed a monodisperse population of particles, with mean volume amounts V = 140 nm^3^, as determined by high resolution AFM. The volume equaled a sphere of a diameter d = 6.4 nm, as expected for CALX-173-GK micelles^27^. This is consistent with AUC data (Table S9). The particles measured on the surface of mica were not spherical. In fact, their lateral diameter is at least twice their maximal height (Fig. 4Aa–c). This might be due to surface wetting and/or mechanical deformation due to the raster scanning process. Both effects are typical for a fluid particle.

In contrast, samples which are loaded with a minute amount of CD20 of 750 ng/ml concentration; showed a bimodal size distribution (Fig. 4Ab,e), and became, at the concentration of 1500 ng/ml, widely monodisperse. A discrete fraction of uniformly larger particles can then be noticed (Fig. 4Ab,c). At a concentration of 1500 ng/ml, individual objects started crowding (Fig. 4Af), forming discrete chains made by two, three or more particles. Interestingly, these particle chains were not necessarily fused; they rather retained their globular discrete shape, as illustrated by the height distribution profiles, and the AFM height maps (Fig. 4Af). The crowding lead to a volume distribution profile which appeared continuous and wide. The CD20 particles, single objects and, also, those assembled in chains, are consistent with well-proportioned flexible particles, without any indication of aggregation by particle fusion. The observation is indicative of discretely dissolved and stabilized protein in a fluid-micellar fashion. No indication for a structured protein was reported so far. We also used negative staining transmission electron microscopy (EM) to compare the particle morphology of micelles loaded with CD20 to empty detergent micelles. Multivariate analysis of particle ensembles picked from raw micrographs in the presence of CD20 gave class averages which reveal roundish and elongated particles. They had a diameter larger than observed with empty detergent micelles (Fig. 4B). The class averages confirmed the flexible nature of the particles; motifs which would be indicative for a distinct structure or protein topology have not been observed.

### Antibody binding to native CD20

**Figure 5:**
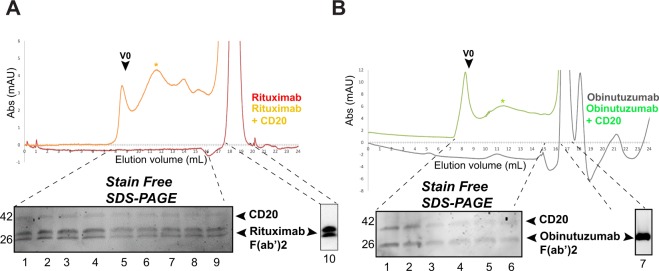
Rituximab and obinutuzumab binding to purified CD20. (**A**) Size exclusion chromatography of CD20/ rituximab complex Top: Gel filtration chromatograms of CD20/ rituximab assembly and rituximab alone are shown in orange and red, respectively; Bottom: SDS-PAGE and stain free detection was used to analyze the gel filtration fractions. *Indicates CD20/ rituximab complex elution. (**B**) Size exclusion chromatography of CD20/ obinutuzumab complex *Top*: Gel filtration chromatograms of CD20/ obinutuzumab assembly and obinutuzumab alone are shown in green and black, respectively; Bottom: SDS-PAGE and stain free detection was used to analyze the gel filtration fractions. *Indicates CD20/ obinutuzumab complex elution. Full-length gels, blots and chromatograms are included in a Supplementary Information file (Figs S2 and S4–8).

To better study CD20/antibody binding in solution, rituximab F(ab’)2 fragment and His-affinity purified CD20 were incubated at 4 °C, concentrated and then loaded on a Superose 6 column. The rituximab fragment alone was also injected on the same column to determine its elution volume. Both chromatograms were superimposed in Fig. 5A. When injected alone, rituximab eluted at 18.5 ml (Fig. 5A top, red curve), its detection on reduced SDS-PAGE showed the presence of mainly two main bands at ~30 kDa (Fig. 5A bottom,) corresponding to the light and cleaved heavy chains. Looking at the CD20/rituximab complex chromatogram (Fig. 5A top, orange curve), we clearly observed an additional peak (indicated with an orange star) at an elution volume of 11.5 ml which was different from the rituximab elution volume (18.5 ml) and the CD20 elution volume (14 ml) shown in Fig. 3A (indicated by an Asterix). Elution fractions of this peak when analyzed by SDS-PAGE could confirm the presence of both CD20 and rituximab **(**Fig. 5A bottom). In contrast to CD20 alone (Fig. 3A), a peak at the void volume of the column was observed which contained also CD20 and rituximab, which most probably corresponds to partially aggregated CD20-antibody complexes. The same result was obtained for CD20/obinutuzumab complex (Fig. 5B). Therefore, SEC shows that native purified CD20 preserves its binding properties to rituximab and obinutuzumab.

**Figure 6:**
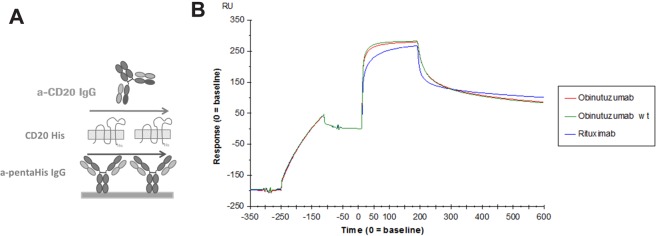
CD20/antibodies binding by SPR. (**A**) Surface plasmon resonance setup. (**B**) Sensorgram overlays showing the binding of anti-CD20 rituximab (blue), obinutuzumab (red) obinutuzumab wt (green) to purified CD20 captured by anti-His antibody.

**Figure 7:**
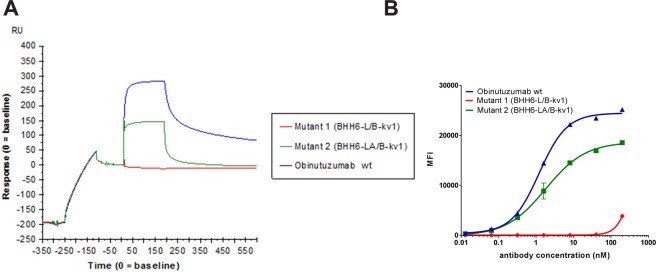
Differential binding of anti-CD20 obinutuzumab and mutants thereof to CD20. (**A**) Surface plasmon resonance sensorgram overlays showing binding of obinutuzumab wt (blue), mutant 1 (BHH6-LA/B-kv1) (red) and mutant 2 (BHH6-L/B-kv1) (green) to purified CD20 (400 nM). For setup, please refer to Fig. 5A. (**B**) Binding to CD20 expressing WSU DLCL2 cells by flow cytometry of obinutuzumab wt (blue), mutant 1 (BHH6-LA/B-kv1) (red) and mutant 2 (BHH6-L/B-kv1) (green).

To further characterize CD20/antibody binding, we applied Surface Plasmon Resonance (SPR). We took advantage of the His-tag of CD20 for immobilization by anti-His specific IgG on SPR chip. Purified full length and wild type CD20 could be effectively captured and anti-CD20 antibodies were allowed to bind (Fig. 6A). Purified CD20 was recognized by rituximab, obinutuzumab and obinutuzumab featuring wild type (wt) glycoforms (Fig. 6B**)**. No significant difference in binding between the three anti-CD20 antibodies was observed under these conditions (Fig. 6B). To confirm the specificity of the binding between obinutuzumab and the purified CD20, series of obinutuzumab mutants were prepared. Since the glycoforms linked to Asn297 of the antibody did not affect binding to CD20 (Fig. 6B, obinutuzumab wild-type and mutants^15^), the mutant were produced with a wild type glycoprofile and compared to obinutuzumab wt. Mutations aimed at reducing or diminishing the binding to CD20 were introduced in the complementarity determining regions (CDRs) of obinutuzumab. Mutant 1 (BHH6-L/B-kv1) was designed to not bind CD20 and mutant 2 (BHH6-LA/B-kv1) was selected as variant with reduced binding to CD20. As can be observed in the SPR sensorgram of Fig. 7A, the extent of binding reflected the different affinities of the anti-CD20 antibody variants. This was confirmed by flow cytometry (FACS), where mutant 1 (BHH6-LA/B-kv1) did not bind to CD20 positive B cell lymphoma WSU DLCL2 cells using concentrations of up to 200 nM (Fig. 7B) and mutant 2 (BHH6-L/B-kv1) showed reduced binding to CD20 when compared to obinutuzumab wt. The use of the obinutuzumab mutants confirmed the specificity of the binding to purified native CD20.

## Discussion

Not much is known about the molecular organization and function of CD20. It is known to have 4 transmembrane domains, and it was suggested to form tetramers^4,29^. Here we show that a purified and non-aggregated form of native CD20 can be obtained from plasma membranes of HEK293 cells as well as cancer cells (SUDHL4 and RAMOS). Negative stain Transmission Electron Microscopy (NS-TEM) and AFM found CD20 in distinctly sized micellar nanoparticles of partly fluidic nature. The particles are flexible and wet the surface of glow- discharged carbon as well as surface of mica, both substrates are mandatory for NS-TEM and AFM experiments. Interestingly, the micellar solubilized CD20 particles do not fuse or aggregate; but remain distinctly proportioned objects, even if the surface is crowded with particles. Both, NS-TEM and AFM, did not reveal rigid monomeric motifs, the particles appear to be droplets. However, it is fair to mention that both methods inspect surface bound states, which is acceptable for many structured proteins but not necessarily valid for all. Therefore, we characterized solubilized CD20 at higher concentrations, in a hydrodynamic environment by means of AUC, and were then able to follow CD20 oligomerization, in a concentration dependent manner. Taken together it can be stated that the applied biophysical methods confirm a fusion stable and flexible micellar CD20 elementary particle, which behaves dynamically and oligomerizes in a concentration dependent manner. This oligomeric state requires further exploration to better understand the distinct role of each of CD20 populations monomeric or oligomeric. It was previously suggested that CD20 may undergo conformational changes from a closer to an open conformation^16^. Further structural characterization is required to better understand the molecular assembly and conformational states of CD20. Revealing the molecular architecture of Type I/II antibodies such as rituximab and obinutuzumab in complex with CD20 is critical for antibody discovery. To achieve this, it is important to isolate the entire CD20 protein without truncation and/or mutations. Previous studies using cyclopeptide/ antibody interactions did help to understand distinctive recognition of the two antibodies to CD20, since they both bind a very proximal epitope in the large loop of the CD20 extracellular domain but in different orientation^16^. We postulate that post-translationally modified protein may reflect as closely as possible the native state. This is why in this study CD20 was produced in eukaryotic cells, whereas the previously reported sources of CD20 were either CD20 peptides^16,30,31^ or the full length version expressed on the inner membrane of *E.coli* membranes^18,32^.

Using calixarene based detergent to solubilize and stabilize oligomeric membrane protein was reported as a successful strategy and an important alternative to refolding, mutagenesis and truncation^20–22,24^. Recently, this approach was used to reveal the functional architecture of a membrane protein transporter KCC2^28^ or to study fragment binding of a GPCR^23^. Here we report the use of the same strategy to isolate full-length, unmutated, homogenous and non-aggregated CD20.

Finally, purified CD20 could bind to rituximab and obinutuzumab as demonstrated by SEC and SPR. This was confirmed by binding of CD20 antibody mutants of different affinities on human B-cell lymphoma cells. SPR shows specific antibody binding to purified CD20 even in absence of any detergent in the running buffer, probably due to the remaining stabilizing calixarene based detergent bound to the protein. The effect of the buffer composition on the binding of CD20 to different classes of antibodies using SPR needs to be further explored side by side with cryo-electron microscopy. Obviously, while demonstration of binding of CD20 antibodies to the extracellular portion is important, it does not necessarily confirm that the full-length protein shows natural functionality/native conformation.

To provide a complete molecular picture of antibodies/CD20 binding and finally to correlate that to diverse cellular responses elicited by different CD20 classes of antibodies, the ultimate goal will be the structural investigation using a native CD20 preparation. Moreover, investigating simultaneous binding of CD20 antibodies to CD20 in solution using a combination of SPR, cryo-EM and analytical ultracentrifugation will be of high interest. This will allow to confirm preliminary SPR data showing that the anti-CD20 antibodies were not able to simultaneously bind to CD20 (data not shown). This is in agreement with previously reported cell based^33^ and crystallographic studies^16^. In addition to solubilized and purified CD20, one of the new approaches could be its reconstitution into proteoliposome. This would have the advantage of providing a natural stabilizing lipid environment and at the same time having only CD20 protein present at the surface of the membranes. Analyzing this CD20 preparation in the presence and absence of two type of antibodies using single particle cryo-EM and/or tomography will certainly help to better understand the structural basis of CD20 recognition. This strategy may also help with designing new CD20 antibodies with different mechanism of action. The approach described here might also be used to study binding of cancer related antibodies to other challenging membrane protein targets to support research for indications with unmet medical needs.

## Material and Methods

### Expression of native CD20 from SUDHL4 and RAMOS cells

At reception, cells were placed in RPMI 1640 medium containing either 10 or 20% of FBS for SUDHL4 and RAMOS cells respectively. In culture, SUDHL4 and RAMOS cells presented the expected morphology and doubling time as obtained from the Leibniz Institute (DSMZ). The cultures were grown at 37 °C, 5% CO2, under shaking (Infors incubator, 120 rpm) following the provider instructions. Cells were amplified for 2 months and cells harvested regularly, when a cell density of about 1.5–2 × 10^6^ cells/ml was reached. After 2 months, a quality control was carried out to ensure that the cultures were free of mycoplasma contamination using (MYCOTRACE kit, GE Healthcare). Cells were harvested from culture medium at 1000 g for 5 min in 50 ml sterile tubes and cell pellets were flash frozen in liquid nitrogen and stored at −80 °C until use. Expression was evaluated by western blot.

### CD20 enrichment from SUDHL4 and RAMOS plasma membranes

SUDHL4 membrane preparations containing 2 mg/ml of protein were solubilized in the extraction buffer (20 mM Tris-Cl pH 8.0, 200 mM NaCl, 10% glycerol, 1X protease inhibitor cocktail (Sigma), 1% Fos-Choline 12) to obtain a final volume of 200 µl. Extraction was performed for 2 h at 4 °C under gentle agitation, then submitted to 100000 g centrifugation for 1 h at 4 °C. Pellets and supernatants were separated and kept on ice. CD20 supernatants were diluted at 1:20 with equilibration buffer (50 mM Tris pH7, 6.5 or 6.2 depending on the test, 0.1% Fos-Choline-12 (FC12), in order to obtain the expected pH and a final NaCl concentration of 10 mM. Diluted supernatants were loaded then onto a DEAE resin (200 µl, anion exchange resin, GE Healthcare, ref 17-0709) and mixed under gentle shaking at 4 °C for 30 min to allow CD20 binding. After 30 min, the flow through was recovered, resin was washed 3 times with 5 volumes of an equilibration buffer, and elution was performed by 5 to 6 steps of 5*1 volumes of resin each (NaCl concentrations varying from 100 mM to 1 M).

### Expression of recombinant native CD20

DNA plasmid pETR17026 (Roche Innovation Center Zurich) coding for the full length CD20 protein (UniProt P11836, sequence contained an additional C-terminal His6 tag) was transformed and amplified in Xl1Blue bacteria at 37 °C overnight using LB media. DNA from 1.6 l culture was extracted and purified by GigaPrep following the supplier’s protocol. To assess CD20 expression in HEK293, 6 wells of 35 mm tissue culture untreated dish were seeded with 1.5 ml of HEK293 cells at 2 × 10^6^ cells/ml. Transfection was performed by adding DNA and PEI in a sequential way to each well as specified in the text. Cells were incubated at 37 °C with agitation in a humidified atmosphere of 8% CO_2_. After 2 h, 1.5 ml of Free Style 293 medium was added to each well. At 24 h and 48 h post-transfection, 500 µL aliquot were collected and centrifuged at 1000 g for 5 min at room temperature. Cell pellets were then lysed with 50 µL RIPA buffer (*Sigma-Aldrich*, cat#R0278). 5 µL samples were analyzed by SDS-PAGE and western blot.

### SDS-PAGE & western blot

Samples were denatured and reduced in SDS/TCEP-loading buffer (*Genetech*, cat#ID1614) and separated on a 4–15% acrylamide gel (4–15% Mini-PROTEAN^®^ TGX Stain-Free™ Gel, *Bio-Rad*, cat#456-8086). Migration was performed in Tris-Glycine-SDS buffer (*Euromedex*, cat#EU0510). The acrylamide gel was then activated under UV light with a ChemiDoc™ MP system (*Bio-Rad*, cat#170-8280) for Stain-Free™ detection. Proteins were subsequently immobilized by electro-transfer to PVDF membrane using the trans-blot turbo transfer system (*Bio-Rad*, cat#170-4155). The immunodetection of CD20 protein was performed using the SNAP i.d.^®^ 2.0 protein detection system (*Millipore*, cat#SNAP2MB1) with an anti-His antibody at 3/2000 (Sigma, Cat # *A7058*). CD20 western blot signal from the Pellet (P) and the Supernatant (SN) fractions were quantified using Image Lab 4.1 software from *Bio-Rad* to evaluate the extraction efficacy.

#### Membrane fractionation

The pellets (14 g) from 3 l HEK293 cells expressing the target were thawed on ice for 1 h with TBS (Euromedex Cat#220: TRIS 0,24 M pH7.5- NaCl 1,37 M - KCl 26,8 mM), Protease Inhibitor Cocktail 1 × (PIC, *Sigma-Aldrich*, SIGMA*FAST* Tablets EDTA-Free, cat #S8830) to reach a final volume of 28 ml. Mechanical cell lysis was performed on ice using a BeadBeater homogenizer with 0.1 mm diameter glass beads, which included 5 pulses of 30 s with 2 min pause in between. Membrane fractionation was carried out at 4 °C by sequential centrifugations: 1000 g for 5 min, 15000 g for 30 min, and 100000 g for 45 min.

#### Total membrane protein quantification

Total protein concentration in the plasma and internal membrane fraction (P100000 and P15000, respectively) was determined with the micro BCA protein assay kit (*Pierce*, cat#23235) using bovine serum albumin (BSA, *Pierce*, cat#23209) as a standard.

#### Native CD20 solubilization

158.5 mg of total (internal and plasma) membrane proteins were incubated for 2 h at 4 °C in a final concentration of 2 mg/ml in a buffer containing 1X TBS, 1X PIC, 2CMC CALX-R2 (0.23%). After solubilization, samples were centrifuged at 100000 *g* for 20 min at 4 °C. Total extract, pellets and supernatants were analyzed by SDS-PAGE and western blot as described above.

#### His-tag affinity chromatography

Soluble fraction was loaded on 8 ml of His-Tag affinity resin (NiCl2 (Sigma Cat#339350) loaded on IMAC-sepharose 6 FastFlow resin (GE-Healthcare BioScience AB Cat#2019-11)). After 2 h incubation at 4 °C, resin was washed with 10 Column Volumes (CV) of TBS 1x + 50 mM Imidazole (Santacruz, Cat# Sc-300829) + 2 CMC CALX-R2 (0.23%) + 150 mM NaCl (300 mM final); 10 Column Volumes (CV) of TBS 1x + 50 mM Imidazole + 2 CMC CALX-R2 (0.23%) + 350 mM NaCl (500 mM final); 10 Column Volumes (CV) of TBS 1x + 50 mM Imidazole + 2 CMC CALX-R2 (0.23%) + 150 mM NaCl (300 mM final). Elution was performed with 3 × 1CV of TBS 1x + 500 mM Imidazole + 2 CMC CALX-R2 + 150 mM NaCl (300 mM final). Samples of each fraction were analyzed by SDS-PAGE and western blot as described above.

#### Analytical gel filtration

Talon Elution fractions were concentrated on Amicon Ultra 10 kDa (Merck-Millipore) to 100 µl, then loaded on a Superose^TM^ 6 Increase 10/300 (Bed volume 24 ml, Void volume 9 ml, GE healthcare). Elution was performed with an isocratic gradient at 0.3 ml/min in TBS 1x, CALX-173-GK 2CMC at 4 °C and followed by UV absorbance at 280 nm.

### CD20/antibody F(ab’)2 assembly

2 ml of the elution 2 fraction (E2) from the CD20 His-affinity purification were incubated with 200 µl of either rituximab or obinutuzumab F(ab’)2 (produced by Roche Innovation Center Zürich, Switzerland), concentrated on Amicon ultra 50 kDa to 100 µl then loaded on Superose 6 10/300 column as mentioned above.

### Atomic force microscopy (AFM)

#### Sample preparation

A circular mica disc (V1, PLANO GmbH, Wetzlar, Germany) with a diameter d = 10 mm was mechanically cleaved with a tweezer. A freshly exposed mica surface was incubated with 60 µl sized droplet of CD20 in the following buffer-detergent system: tris(hydroxymethyl)-aminomethan (Tris), c = 20 mM; pH = 8; sodium chloride, c = 300 mM; imidazole, c = 500 mM; CALX-173-GK, c = 3 mM. Three different protein incubation concentrations were selected to adjust and vary particle surface coverage: c_i_ = 0, 0.75, 1.5 µg/ml. After 1.5 minutes the incubation liquids were removed, and the surfaces washed fourth times with fresh buffer.

#### AFM experiment

The samples were mounted on sample stage of an Atomic Force Microscope (FastScan ICON, Nanobruker, Santa Barbara, United States) to be analyzed with tapping mode in liquid (tris(hydroxymethyl)-aminomethan (Tris), c = 20 mM; pH = 8; sodium chloride, c = 300 mM; imidazole, c = 500 mM; CALX-173-GK, c = 3 mM), a well calibrated scanner was used. A small and sharp AFM cantilever (FastScan DSS, Nanobruker, Camarillo, United States) with a nominal tip radius of r ~ 1 nm, a nominal resonance frequency ν ~ 110 kHz in liquid, a nominal force constant f = 0.25 N/m was used. All samples were scanned with line speed of about between 2 Hz at setpoint amplitudes of A = 3.5 nm, at drive amplitudes corresponding to V = 1500 mV. The integral gain amounted IG ~ 2, and the z-scanner range was Z_range_ = 0.5 µm. 5 × 5 µm^2^ sized regions were analyzed with a spatial resolution corresponding to maximal ΔxΔy = 1 nm^2^, Δz ~ 0.01 nm, more than 1000 particles were inspected.

#### Image processing

All height maps were plane corrected using a 1^st^ order polynomial fitting procedure, a median filter was applied (3 × 3) using NanoScope Analysis, Vers. 1.8 R2Sr2 (Nanobruker, Santa Barbara, United States). The processed data were imported into Scanning Probe Image Processor Vers 6.5.3 (Image Metrology, Horsholm, Denmark, EU). The particle size distribution module was used to determine particle size distribution histograms. To do so, height maps were analyzed with a threshold mask, at z = 0.5 nm (above mean plane). Only objects which occupied an area larger than 20 nm^2^ and were taller than z = 0.5 nm were considered to be a particle. Three different distribution profiles were determined: (i) particle volume distribution weighted by volume, (ii) maximal z height distribution (relation of particle counts), (iii) particle diameter distribution (relation of particle counts). The histograms were displayed with GraphPad Prism Vers 7.03 (Statcon, Witzenhausen, Germany, EU).

### Negative stain electron microscopy

#### Grid preparation

Freshly thawed samples were diluted in detergent containing buffer to c ~ 0.02 mg/ml. 4 µl of the diluted sample or undiluted buffer was adsorbed to glow discharged 400 mesh carbon coated parlodion copper grids washed with 3 drops of water, were incubated with 3 µl of Tobacco Mosaic Virus (TMV) containing solution, further washed with 2 drops of water and finally stained with 2 drops of uranyl acetate 2%.

#### Transmission electron microscopy

Samples were imaged using a Tecnai12 transmission electron microscope (FEI, Eindhoven, The Netherlands) operating at 120 kV. Electron micrographs were recorded on a 4096 by 4096-pixel charge-coupled device camera (TVIPS F416) at a nominal magnification of 52.000x yielding a final pixel size of 0.253 nm on the specimen level.

#### Image processing

Reference-free alignment was performed on manually or automatically selected particles from recorded images using the EMAN2 image processing package. The extracted particles were aligned and classified by multivariate statistical analysis yielding 2D class averages.

### Surface plasmon resonance

The SPR experiment was performed on a Biacore T200 at 25 °C with HBS-EP as running buffer (0.01 M HEPES pH 7.4, 0.15 M NaCl, 3 mM EDTA, 0.005% Surfactant P20, Biacore, Freiburg/Germany). Purified native CD20 was captured by an anti-penta His antibody (Qiagen, Switzerland) immobilized on CM5 chip by amine coupling (immobilization levels approx. 12500 RU). Purified native CD20 was diluted in HBS-EP+ buffer and injected as 400 nM solution for 140 seconds on the flow cells (10 μl/min flow). The anti-CD20 antibodies were injected at a concentration of 5 μM with a flow of 30 μl/min through all flow cells over 180 sec. Dissociation was monitored for 600 sec. The chip surface was regenerated after every cycle using two injections of 10 mM Glycine pH2 for 60 sec. Bulk refractive index differences were corrected for by subtracting the response obtained in a reference flow cell, where no purified CD20 was captured.

### Antibodies binding to cells

1.2 × 10^5^ cells/well of WSU DLCL2 (DSMZ, cultivated in RPMI1640 containing 10% FCS and 1% Glutamax (Invitrogen/Gibco # 35050-038)) were incubated in a 96-U-bottom plate for 30 min at 4 °C with titrations of anti-CD20 antibodies (0.01-200 nM) in FACS Buffer (PBS + 2% FCS + 5 mM EDTA + 0.25% sodium acid). After washing, cells were incubated for an additional 30 min at 4 °C in the dark with a AlexaFluor647-conjugated F(ab)‘2 goat anti-human Fcγ specific secondary antibody (Jackson Immunoresearch). After washing, cells were fixed with 2% PFA in FACS Buffer and measured using a FACSCantoII (BD Bioscience). The average median fluorescence intensity and SDs were calculated in triplicates.

### Analytical ultracentrifugation

Sedimentation velocity experiments were performed at 60’000 rpm in an An-60 Ti rotor on a XLI instrument (Beckman Coulter, CA, USA) at 20 °C with SedVel60k (SpinAnalytical, ME, USA) centerpieces. The sedimentation process was monitored with interference or absorbance detection at 280 nm. CD20 protein was measured at 0.5 mg/ml in CALX-173-GK containing buffer (20 mM Tris, pH 8.0, 150 mM NaCl, 0.05 mM CALX-173-GK (0.0072%)) or at 0.044 mg/ml after dilution with CALX-173-GK or DDM carrying buffer (20 mM Tris, pH 8.0, 150 mM NaCl, 0.05% DDM).

### Evaluation of sedimentation data

AUC velocity sedimentation data were evaluated with Sedfit as previously reported^34^ to obtain sedimentation coefficient distributions c(s) using sedimentation coefficient distribution c(s) with a single frictional ratio for all sedimenting species. Partial specific volume of CD20 protein (0.741 ml/g) was calculated from protein amino acid sequence. Sedimentation coefficient distributions c(s) were superimposed using GUSSI software (version 1.0.8e)^35^.

### Density and refractive index measurements

Density and refractive index measurements for CALX-173-GK detergent as function of detergent concentration in Tris buffer (20 mM Tris, 150 mM NaCl) were performed on density meter DMA5000M (Anton Paar, Graz, Austria) and viscometer AMVn (Anton Paar, Graz, Austria) at 20 °C.

### Disclosures

All Roche authors declare employment with Roche as well as stock ownership. University of Basel receives funding from Roche. The authors MA, EM, TI & AJ are employees of CALIXAR that have patents applications that covers some of CALX detergent described in this manuscript. CALIXAR received funding from Roche for part of the reported work.

## Supplementary information

Supplementary Information
