# Supplementary material for: Biochemical and biophysical characterization of purified native CD20 alone and in complex with rituximab and obinutuzumab
Source: Sci Rep. 2019 Sep 23;9:13675. doi: 10.1038/s41598-019-50031-4 (PMC6757138; doi:10.1038/s41598-019-50031-4)
Supplement: Supplementary file 1 — Supplementary Information [file 41598_2019_50031_MOESM1_ESM.pdf]

SUPPLEMENTARY MATERIAL

TITLE: Biochemical and biophysical characterization of purified native CD20 alone and in complex with rituximab and obinutuzumab

AUTHORS:

Morgane AGEZ[1], Elodie DESUZINGES MANDON[1], Thomas IWEMA[1], Reto GIANOTTI[2], Florian LIMANI[2], Sylvia HERTER[2], Ekkehard MÖSSNER[2], Eric A KUSZNIR[3], Sylwia HUBER[3], Matthias LAUER[3], Philippe RINGLER[4], Claudia FERRARA[2], Christian KLEIN[2] & Anass JAWHARI[1¶]

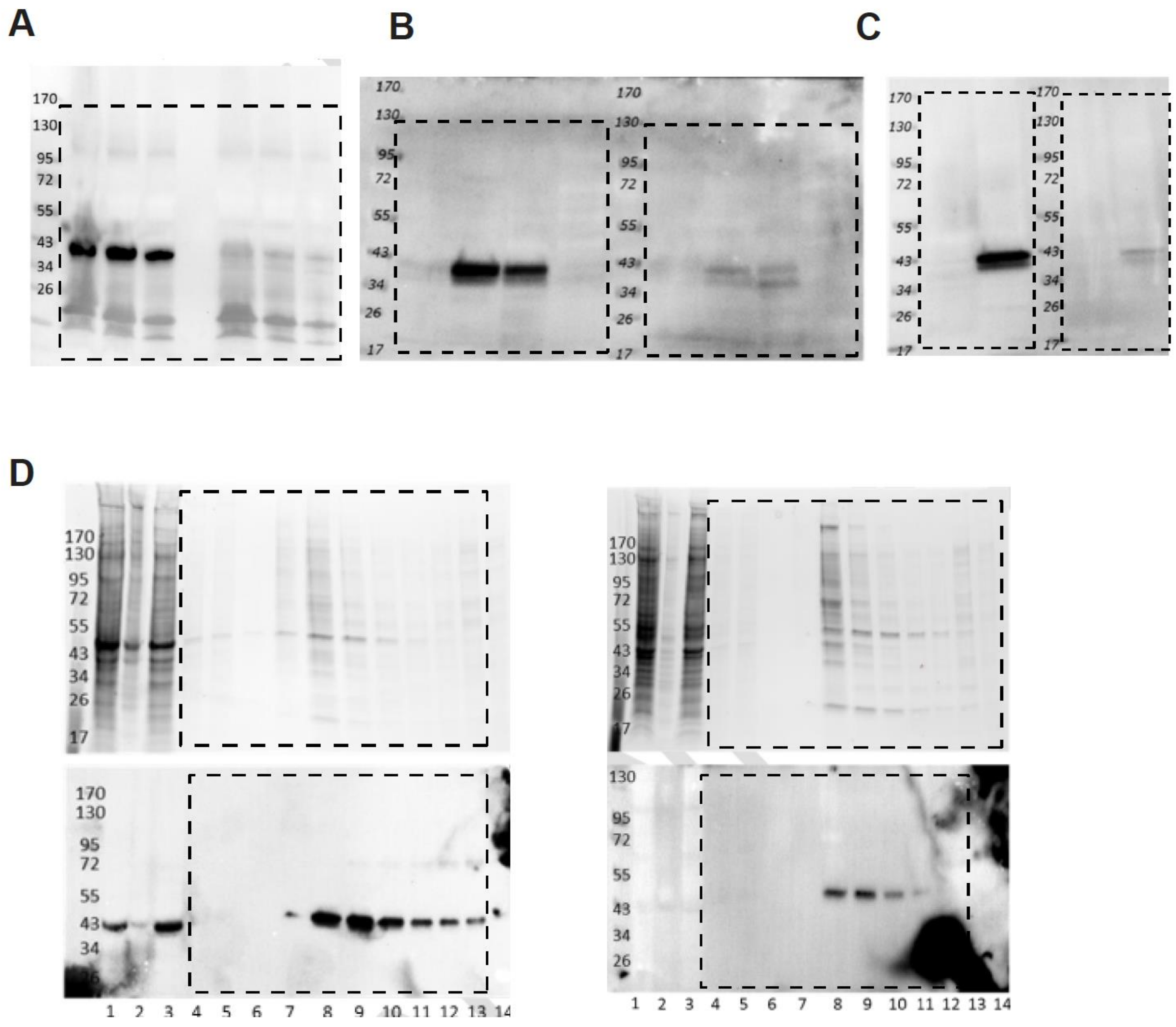

Figure S1: Original gels or Western-blot used for Figure 1A-D. A rectangle represents what is shown in the main figures.

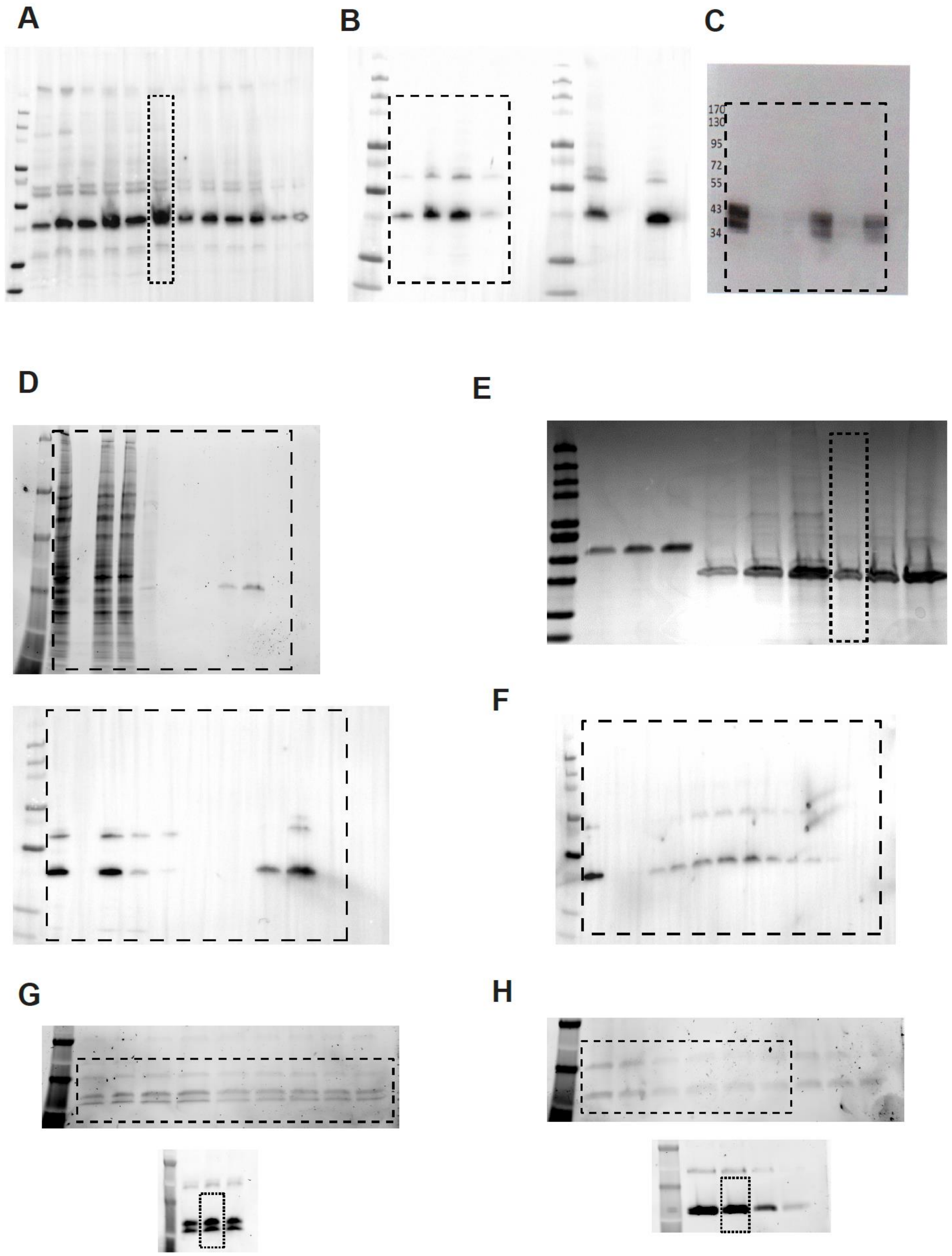

Figure S2: Original gels or Western-blot used for the main Figures. A-E correspond to Figure 2A-E. F corresponds to Figure 3A. G-H correspond to Figure 5A-B. A rectangle represents what is shown in the main figures.

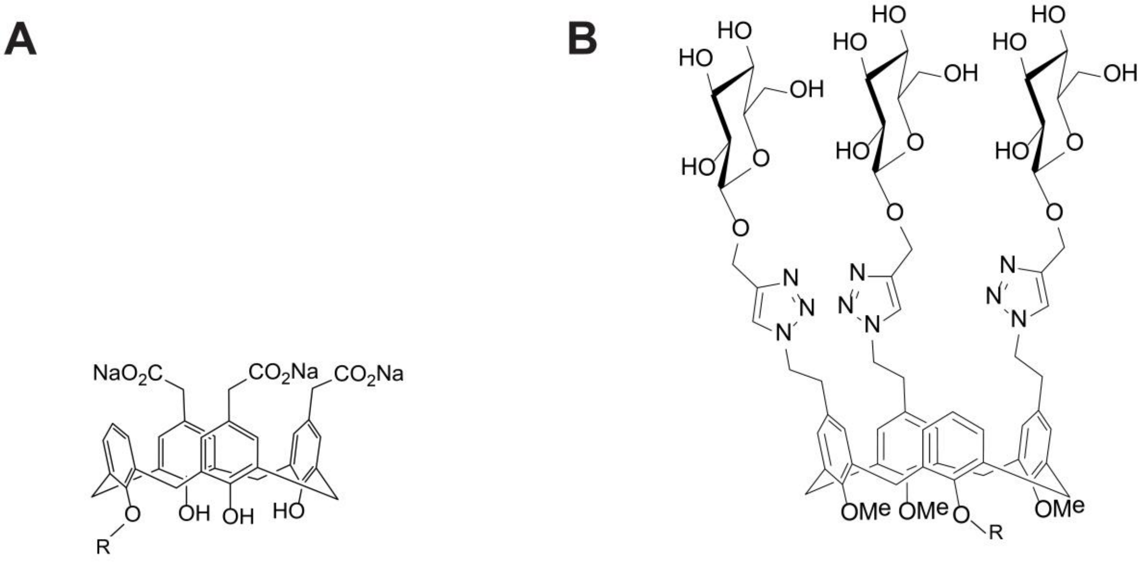

Figure S3: Structure of two calixarene based detergents used for solubilization and purification of CD20. **A-** 5,11,17-tris[(carboxy)methyl]-25-monooctyloxy-26,27,28-trihydroxycalix[4]arene. Also called CALX-R2 in this manuscript. **B-** 5,11,17-tris(2-[4-(β-D-glucopyranosyl)-1H-1,2,3-triazol-1-yl]ethyl),-25- monoheptoxy-26,27,28-trimethoxycalix[4]arene; respectively, also called CALX-173-GK. R corresponds to an aliphatic tail of seven carbons.

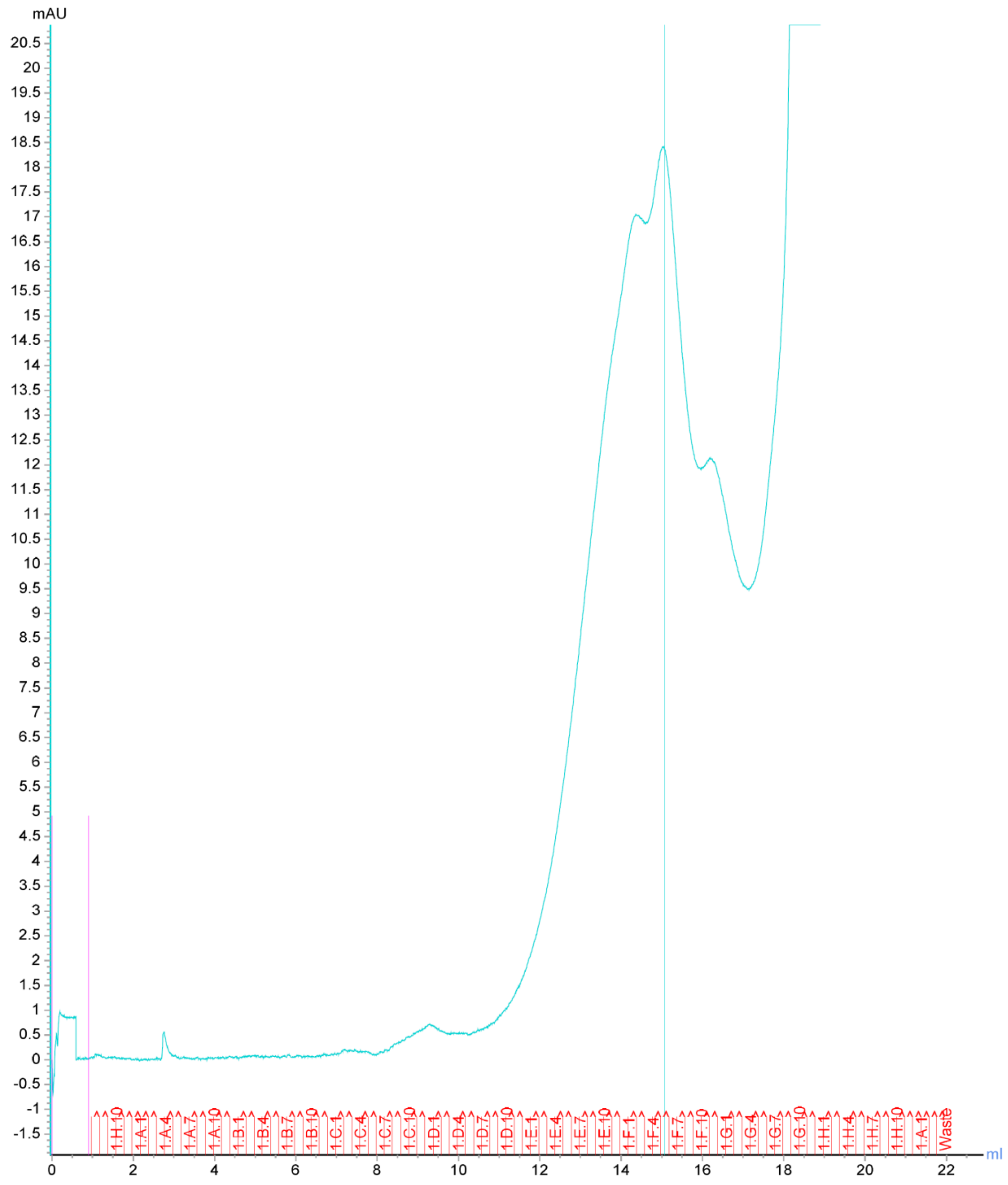

Figure S4: Full length size exclusion chromatogram of purified CD20.

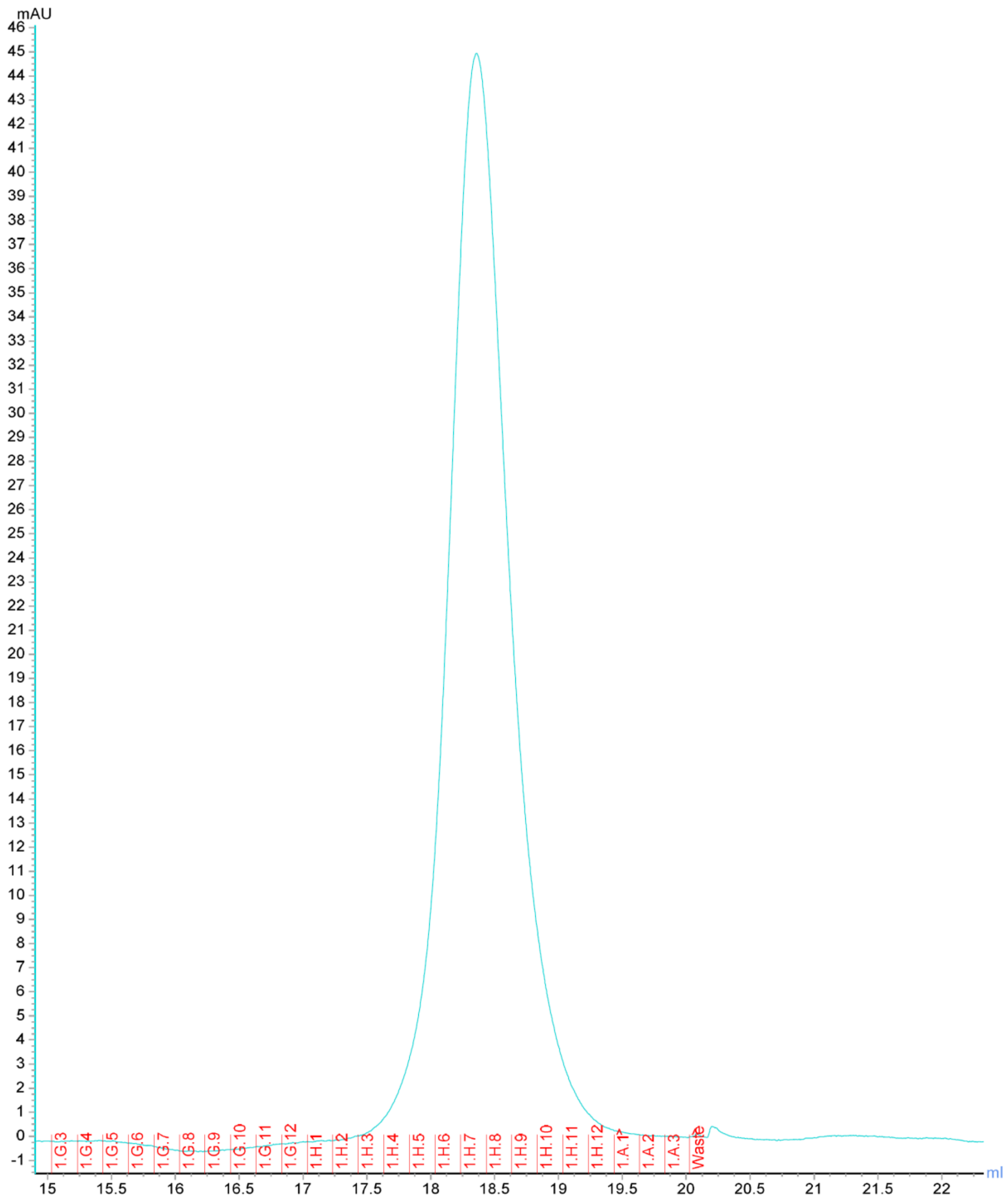

Figure S5: Full length size exclusion chromatogram of the rituximab.

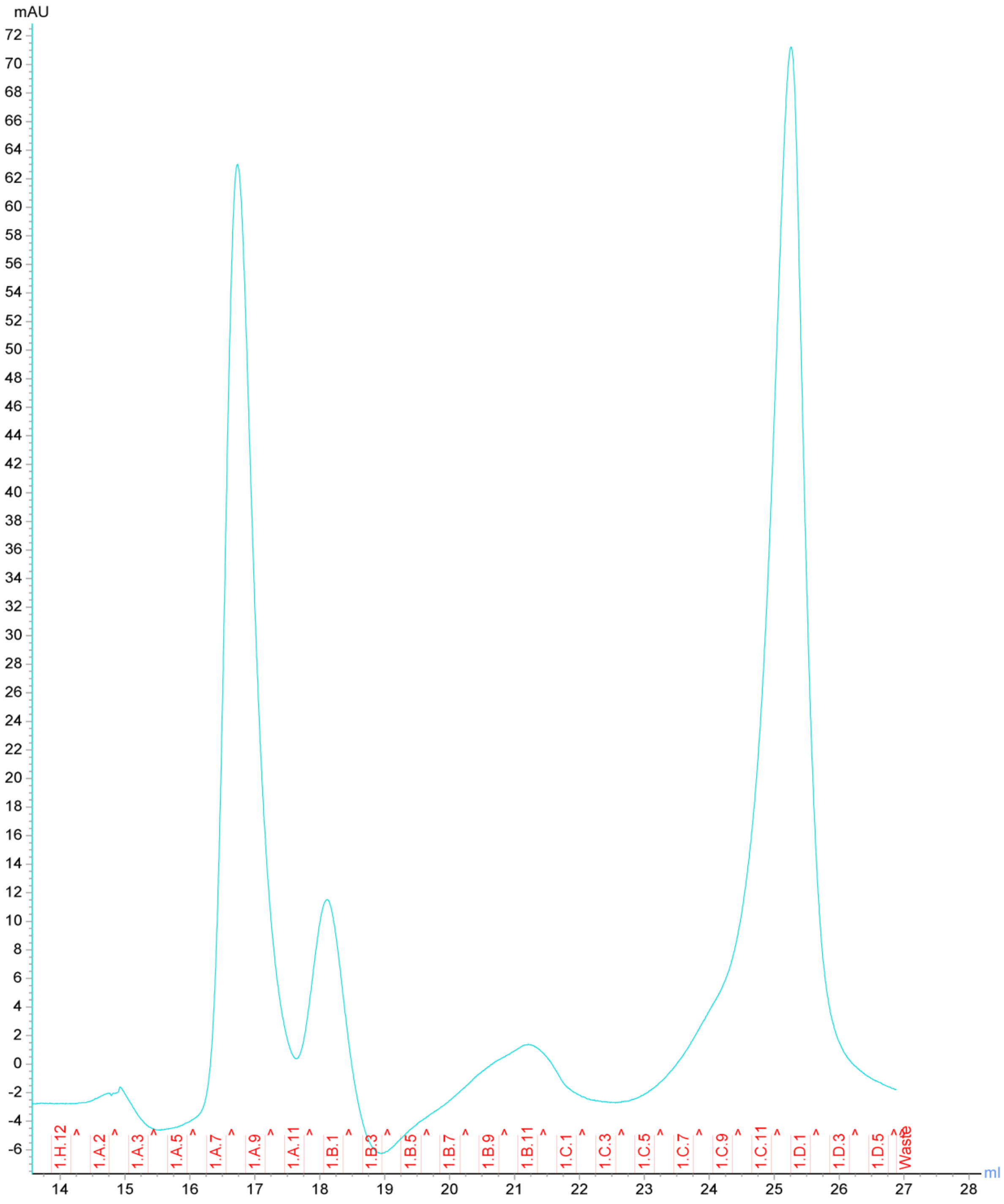

Figure S6: Full length size exclusion chromatogram of the obinutuzumab.

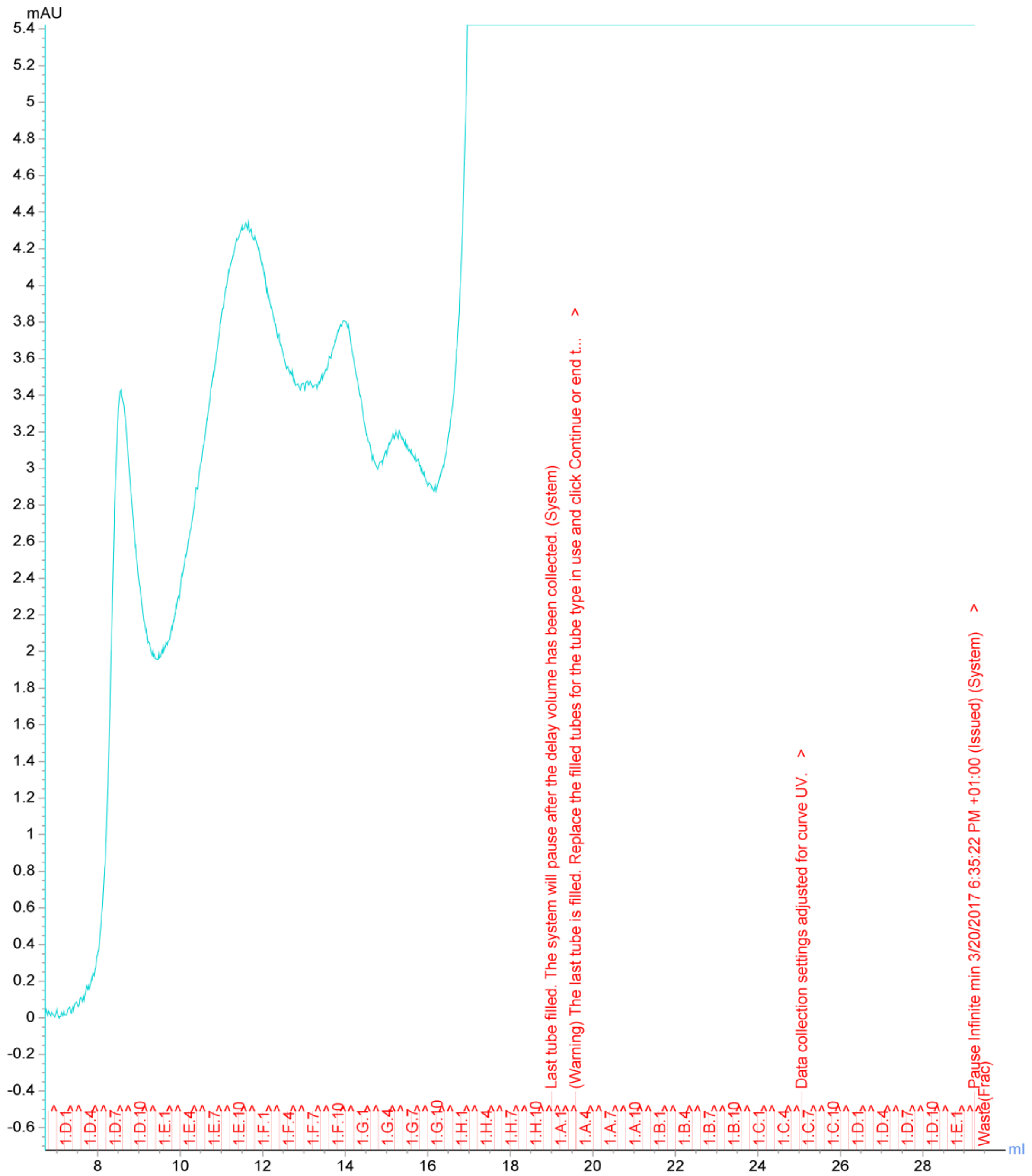

Figure S7: Full length size exclusion chromatogram of CD20/rituximab complex.

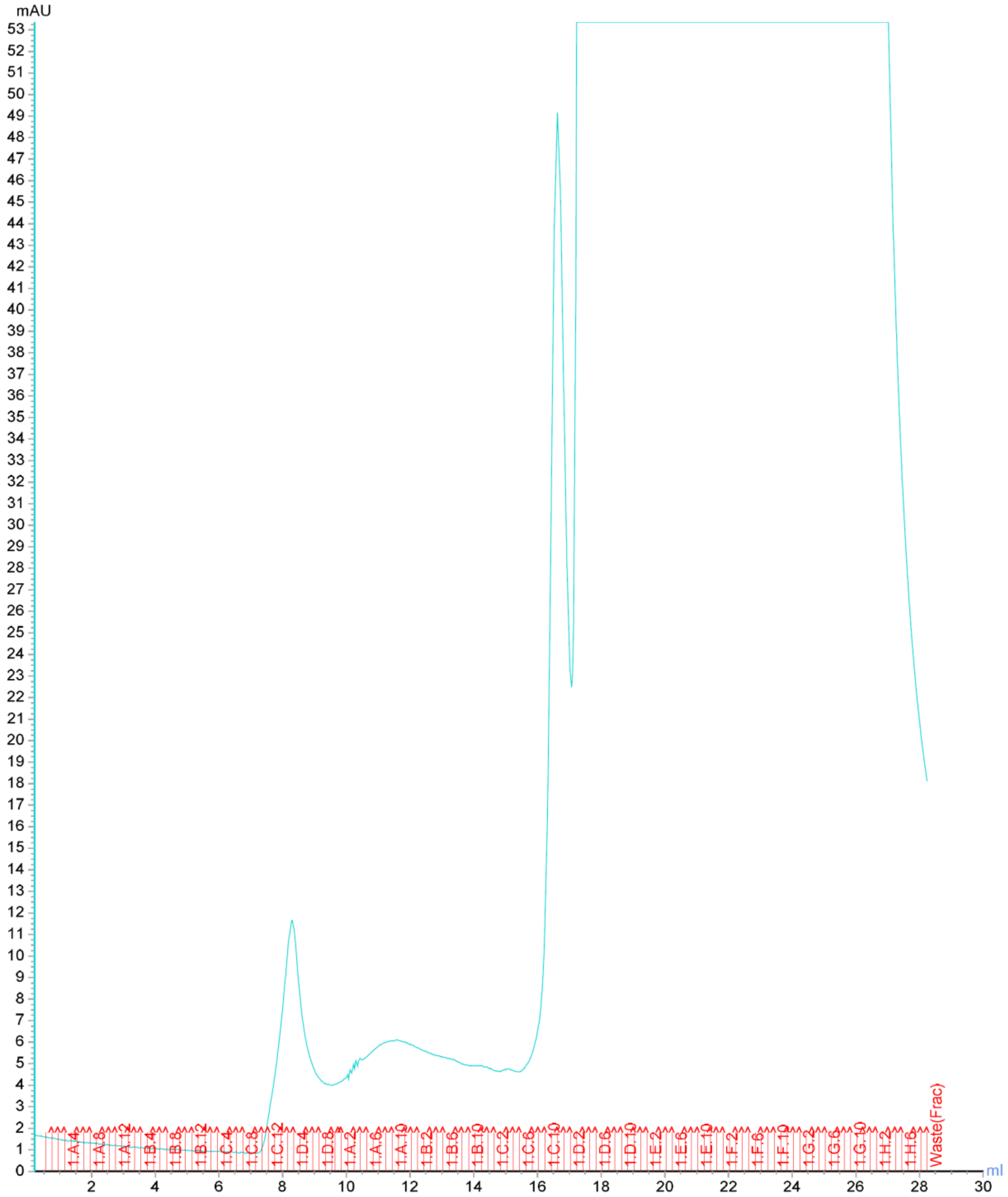

Figure S8: Full length size exclusion chromatogram of CD20/ obinutuzumab complex.

Sample	conc. [mg/ml]	Detergent in buffer	Detection system	Peak	sw [S]	f/f0	Area [%]	vbar [ml.g^{-1}]	$MW_{expec.}$ [kDa]	$MW_{calc.}$ [kDa]	Interpretation
CALX-173-GK alone	5,000	CALX-173-GK	Interference	1	2,9	1,18	100	0,764	no	38	detergent micelle
CD20	0,500	CALX-173-GK	Absorbance	1	3,5	1,35	30	0,741	34	~ 54	probably monomeric CD20
				2	4,6		70			> 80	higher oligomers
				3	5,8						
				4	7,7						
				5	10,0						
CD20	0,500	CALX-173-GK	Interference	1	3,5	1,35	27	0,741	34	~ 61	probably monomeric CD20
				2	4,4		73			> 84	higher oligomers
				3	5,8						
				4	7,4						
				5	8,6						
				6	9,9						
DDM alone	20,000	DDM	Interference	1	2,9	1,00	100	0,836	50	53	detergent micelle
CD20	0,044	CALX-173-GK	Absorbance	1	3,6	1,35	46	0,741	34	~ 57	probably monomeric CD20
				2	5,8		17			>100	higher oligomers
				range	6-12		37				
CD20	0,044	DDM	Absorbance	1	3,1	1,42	72	0,741	34	~ 48	probably monomeric CD20
				2	5,0		28			99	probably dimer

Table S9: Summary of AUC calculations of the molecular mass of CD20 species.

Expected molecular mass (MW_{expec}) was calculated from protein amino acid sequence. The partial specific volume (vbar) of the protein alone was taken into account as it is impossible to determine exact detergent/protein ratio due to simultaneous absorbance of the protein and CALX-173-GK detergent. Therefore, the calculated molecular masses should be interpreted as “estimates”.
